# Enhanced Drive Current in 10 nm Channel Length Gate-All-Around Field-Effect Transistor Using Ultrathin Strained Si/SiGe Channel

**DOI:** 10.3390/mi15121455

**Published:** 2024-11-29

**Authors:** Potaraju Yugender, Rudra Sankar Dhar, Swagat Nanda, Kuleen Kumar, Pandurengan Sakthivel, Arun Thirumurugan

**Affiliations:** 1 Department of Electronics and Communication Engineering, National Institute of Technology Mizoram, Aizawl 796 012, Mizoram, India; p.yugender@gmail.com (P.Y.); nanda.swagat@gmail.com (S.N.); kuleen.elx@gmail.com (K.K.); 2 Centre for Materials Science, Department of Science and Humanities-Physics, Faculty of Engineering, Karpagam Academy of Higher Education, Coimbatore 641 021, Tamil Nadu, India; 3 Sede Vallenar, Universidad de Atacama, Costanera 105, Vallenar 1612178, Chile; arunthiruvbm@gmail.com

**Keywords:** GAAFET, heterostructure-on-insulator, on current, stacked high-K, strained silicon

## Abstract

The continuous scaling down of MOSFETs is one of the present trends in semiconductor devices to increase device performance. Nevertheless, with scaling down beyond 22 nm technology, the performance of even the newer nanodevices with multi-gate architecture declines with an increase in short channel effects (SCEs). Consequently, to facilitate further increases in the drain current, the use of strained silicon technology provides a better solution. Thus, the development of a novel Gate-All-Around Field-Effect Transistor (GAAFET) incorporating a strained silicon channel with a 10 nm gate length is initiated and discussed. In this device, strain is incorporated in the channel, where a strained silicon germanium layer is wedged between two strained silicon layers. The GAAFET device has four gates that surround the channel to provide improved control of the gate over the strained channel region and also reduce the short channel effects in the devices. The electrical properties, such as the on current, off current, threshold voltage (V_TH_), subthreshold slope, drain-induced barrier lowering (DIBL), and I_on_/I_off_ current ratio, of the 10 nm channel length GAAFET are compared with the 22 nm strained silicon channel GAAFET, the existing SOI FinFET device on 10 nm gate length, and IRDS 2022 specifications device. The developed 10 nm channel length GAAFET, having an ultrathin strained silicon channel, delivers enriched device performance, being augmented in contrast to the IRDS 2022 specifications device, showing improved characteristics along with amended SCEs.

## 1. Introduction

The semiconductor industry is facing major difficulties in manufacturing faster and denser devices, which proves a challenge. The channel length of the device keeps decreasing continuously in MOSFET fabrication. In shorter lengths below 14 nm, a single gate or two gates are not sufficient, making it more challenging to sustain control over the channel. This causes short channel effects (SCEs) [1,2], such as impact ionization, velocity saturation, surface scattering, and drain-induced barrier lowering (DIBL) [3]. When drain voltage (V_DS_) increases, i.e., V_DS_ >> V_GS_, the small channel barrier from the drain end is lowered, and the charge carrier can be surpassed easily at low gate voltage, referred to as DIBL. When the electrons are accelerated towards the interface, collisions will occur, which are called surface scattering [4]. In saturation mode, the transconductance is reduced due to velocity saturation. Electron–hole pairs are generated in the high longitudinal fields as the velocity of electrons is high and the impact ionization affects the silicon atoms. As the V_DS_ becomes high, the electrons move into the channel, which is called the hot electron effect [5].

The current direction is parallel to its wafer, and the channel is aligned vertically in the double-gate FinFET, so it is called quasi-planar. The FinFET has high design space as the gate terminal is shorted or independently controlled. The SCEs are reduced as the channel region is controlled with two gates. Due to strong coupling in thechannel, superior control over the channel was attained with higher noise tolerance and low powerconsumption [6]. As scaling parameters are deployed in FinFET, the SCEs also affect the performance, but still, FinFETs are capable of sustaining design parameters, reducing subthreshold swing and enabling faster switching times.

Manufacturing technology continues to evolve, and FinFET technology has been continuously growing sincethe last decade. However, with continuous downscaling, FinFETs have also reached their minimal V_TH_ level [7,8], making them increasingly difficult to scale down, and their performance efficiency is not significant. In the year 1988, Toshiba introduced the first Gate-All-Around Field-Effect Transistor (GAAFET) [9], fabricated vertically, which was also called a Surrounding-Gate Transistor. A GAAFET works very similarly to a FinFET transistor. In this GAAFET, the gate material occupied the overall surface of the conducting channel. GAAFETs typically have four gates, which provide the best gate controlling ability to control the channel, high performance in smaller devices, and a very good ability to mitigate SCEs with minimal leakage current [10,11]. The Gate-All-Around technology is considered to be the best substitute for FinFETs, because the width of nanosheets can be optimized to enhance packaging density. This gives engineers a wide scope for analzying the characteristics of the device; for instance, a wider blade delivers more current, but the effective cost is increased. In memory cell design, nanosheet (NSH) technology enhances SRAM cell write ability by offering a 50 mV higher write trip point (WTP) compared to FinFET (FF) SRAM. This improvement is attributed to reduced bit line (BL) resistance from a wider metal critical dimension (CD) and over 15% greater drive current strength for the same leakage [11]. If manufacturers wish, they can miniaturize the device to reduce drive current. In addition, GAA technology has provided an alternative for further miniaturization, which was not feasible in FinFET due to design complexity.

GAAFET provides greater controllability over the entire channel so that device performance is enhanced. With scaled channel dimensions and layout, the leakage current is marginal and SCEs are insignificant. In order to keep abreast of the requirements of miniaturization in device feature size specifications [12], the incorporation of strained silicon technology has become inevitable.

Strained silicon technology enhances the mobility of a device, which is achieved by applying an appropriate amount of stress, providing effective mass reduction in the charge carrier in strained regions and leading to enriched driving current at a particular applied gate voltage while keeping the oxide thickness constant. Therefore, the device needs to be developed with thicker gate oxides, allowing the gate voltage to provide a constant drive current and weakening the trade-off relationship between drive current and SCEs, which is beneficial for enhancing device performance [13]. The reductions in the effective mass of the charge carriers and interval phonon scattering per unit time are the primary causes of the improved mobility in strained silicon, which directly increases the device’s driving current, which is the requirement for having faster switching devices [14].

Strain can be incorporated in the device either as local strain or global strain [15,16,17]. Khiangte et al. [18] developed an n-channel MOSFET on buried oxide with a strain Silicon (s-Si) channel to augment the drain current of the nanodevice. Thereafter, Kumar et al. [19] designed the HOI Double-Gate (DG) FET to further control the channel using two gates in the device and attained enhanced performance with optimal SECs, but the device was not capable of sustaining a 10 nm channel length with minimum subthreshold leakage current and high output current.

Therefore, the motivation stands to implement a novel 10 nm channel length GAAFET with a strained silicon channel. In GAAFETs, the gate completely surrounds the channel, providing superior electrostatic control over the channel compared to FinFETs, thereby reducing short-channel effects and leakage currents. When strain is applied to silicon in GAAFETs, it alters the silicon lattice structure, enhancing electron and hole mobility, which results in faster switching speeds and higher drive currents. Strain engineering optimizes this mobility by compressing or stretching the silicon lattice depending on the carrier type, with tensile strain benefiting electron mobility and compressive strain favoring hole mobility. Consequently, integrating strained silicon with GAAFETs enables more efficient, high-performance transistors, particularly advantageous at smaller nodes where space and power efficiency are critical. This GAAFET device is optimized with a 10 nm gate length with three layers comprising (s-Si/s-SiGe/s-Si) in the channel. The various electrical parameters are compared with the 22 nm GAAFET, which makes the device useful for the present technology.

## 2. Device Structure

**Figure 1:**
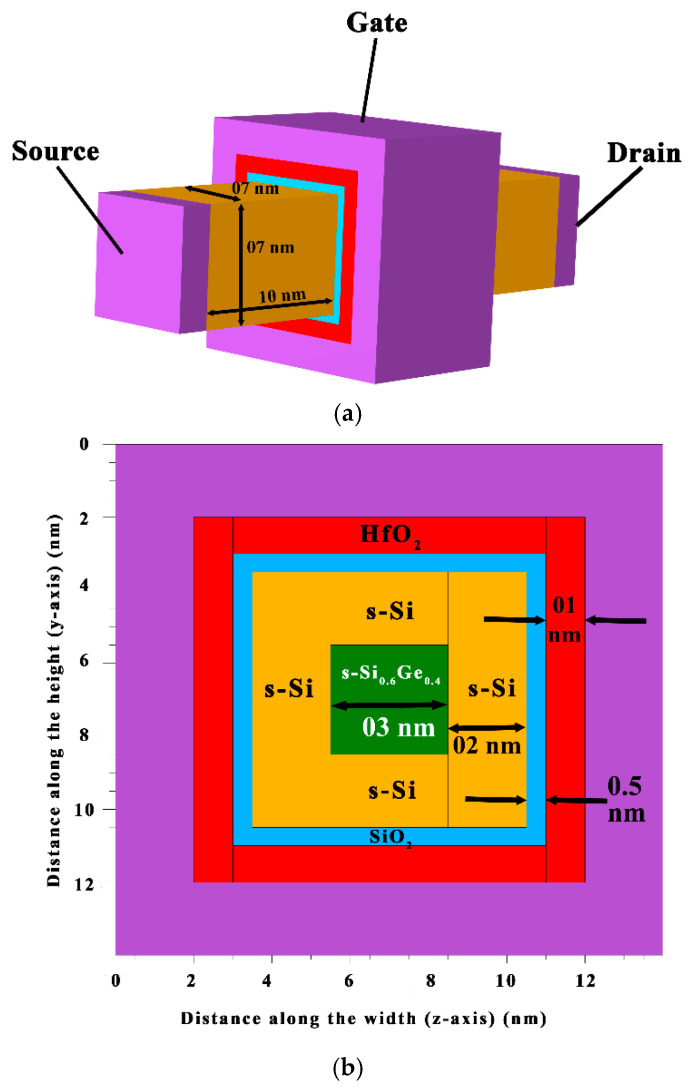
(**a**) Three-dimensional view of 10 nm GAAFET structure. (**b**) Geometrical view of gates all around the channel.

The GAAFET with a strained silicon channel (Si/SiGe) is modelled at a 10 nm channel length, which consists of three layers in the rectangular channel. A 3D view of the GAAFET structure and the measurements of the three layers in the channel are depicted in Figure 1a. The symmetric design is chosen for the source and drain with a 10 nm lateral length. The thickness of three layers is configured with a mole fraction of 40% of germanium in silicon. As the SiGe layer is lowered to ultrathin dimensions of 3 nm, compressive strain is also observed from all sides in the SiGe layer. Therefore, the SiGe layer becomes strained and is, henceforth, abbreviated as s-SiGe in the devices.

The geometric layout of the gates surrounding the channel is illustrated in Figure 1b, showing four gates positioned on each side of the channel. A stacked high-k dielectric with an equivalent oxide thickness (EOT) of 0.7 nm has been selected for the gate oxide, following established and validated studies [12]. This configuration improves channel control. Early studies indicate the integration of high-k dielectric technology with SiO_2_ using EOT [20], significantly reducing scattering. Thus, it is possible to increase the physical thickness of the gate oxide without impacting the calculated capacitance, as follows:

(1) $$EOT=\frac{\varepsilon_{{SiO}_{2}}}{\varepsilon_{high-k}}\times t_{high-k}$$

where $\varepsilon_{{SiO}_{2}}$ is the permittivity value of SiO_2_, $\varepsilon_{high-k}$ is the permittivity value of particular dielectric material, and $t_{high-k}$ is the thickness of material considered in particular device design.

However, with increasing permittivity values, the physical design thickness of the SiO_2_ increased. It is observed that the use of an interfacial SiO_2_ layer decreases the overall physical thickness of the oxide layer [21] while the capacitance remains the same. So, for the gate stack, a combination over the channel region is required, and the EOT [22] is now calculated as:

(2) $$EOT=\frac{\varepsilon_{{SiO}_{2}}}{\varepsilon_{high-k}}\times t_{high-k}+t_{{SiO}_{2}}$$

where $t_{{SiO}_{2}}$ is the thickness of the SiO_2_ layer, with added dielectric material and other parameters considered the same as Equation (1). 

The device design criteria of the 10 nm GAAFET are tabulated in Table 1. The nanostructure device for fabrication is segmented into different parts with respect to the materials required. The silicon is used as a dopant and a combination of SiO_2_ and HfO_2_ as a gate dielectric in the device. The channel region is initially grown with an epitaxial process forming the bottom s-Si layer, and the s-Si_1−x_Ge_x_ layer is formed over this with a mole fraction of 0.4. The Si_0.6_Ge_0.4_ layer is then selectively etched from the sides using lithography and masking. The sides are then infused with s-Si regions to form the back and front s-Si regions. The top s-Si layer is then epitaxially grown to complete the three-layered strained nanosystem. In the next step, a pentavalent impurity is added to the silicon region, which serves as the device’s source, and drains are formed in symmetrical nature, therefore developing the strained channel rectangular GAAFET.

The dopant atom concentration in the source and drain is 5 × 10^19^ cm^−3^, respectively, while the channel is doped with an acceptor impurity of 1 × 10^15^ cm^−3^ with consideration of the dielectric material work function, which is 4.6 eV. This device is implemented using Silvaco TCAD [23]. The Shockley Read Hall model, the Hansch quantum effects model, the BGN model and the Auger recombination model [23] are integrated into the simulations to impart a correct understanding of the developed device.

## 3. Results and Discussion

The initial rectangular GAAFET with a strained silicon channel is developed on a 10 nm channel length with a tri-layered channel of 2 nm, 3 nm, 2 nm, named device A, where the gate oxide is 1 nm of SiO_2_. These electrical properties of device A are compared to the 22 nm channel length device GAAFET, which consists of a strained silicon channel with three layers of s-Si, s-SiGe, s-Si and channel thickness 2 nm, 4 nm, 2 nm, respectively [24], termed device B, a recent silicon channel GAAFET of gate length 22 nm, named as device C [24], a 10 nm Silicon On Insulator (SOI) Trigate (TG) FinFET, named as device D [25], and IRDS 2022 specifications, named as device E [12]. Device A is further modified where the gate oxide encompassing the channel is a stacked combination of SiO_2_ and HfO_2_ to have a combined EOT of 0.7 nm and named device F.

The details of all the devices are provided in Table 2. From the electrical properties, the optimum device has been analyzed with minimum SCEs at a 10 nm gate length.

**Figure 2:**
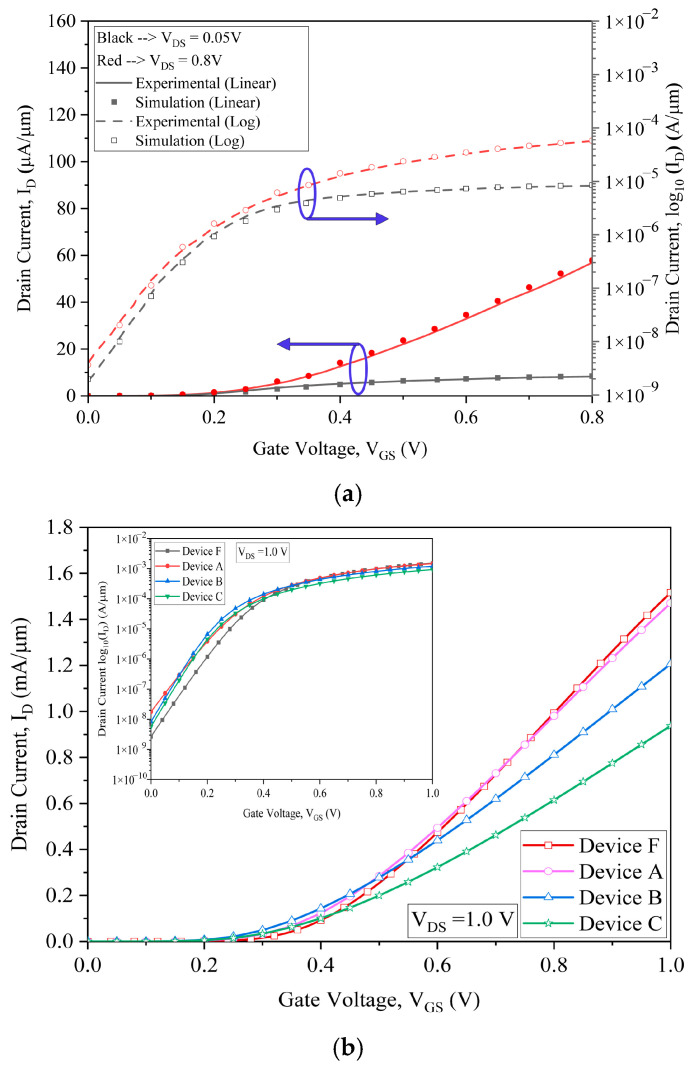
(**a**) Calibration of simulation results with experimental data [26], (**b**) linear and logarithmic transfer characteristics of device A, B, C and F.

The Silvaco Atlas simulator calibration is carried out with simulated I_D_–V_GS_ plots from the experimental data of Lin et al. [26]. The I_D_–V_GS_ of the developed device by Lin et al. [26] is generated almost accurately, as reported and shown in Figure 2a. The current and voltage linear characteristics of device A, device B, and device C of the GAAFETs are plotted in comparison to that of device F at a drain voltage of 1 V, and a comparison graph is shown in Figure 2b, which clearly depicts that device F has the highest on current and least leakage current among all devices, which will also have fewer short channel effects. The drain current is almost increased by 60% when compared with device F. As we induced the tri-layered channel, the device output current increased with less leakage current. The logarithmic characteristics are shown in the inset in Figure 2b. 

As the device performance is affected by short channel effects for which V_DS_ is crucial for changing the $V_{TH}$, the $V_{TH}$ is expressed in (3) [27] as

(3) $$V_{TH}=\emptyset_{GC}-{2\emptyset }_{F}-\frac{Q_{B}}{C_{ox}}-\frac{Q_{ox}}{C_{ox}}$$

where ∅*_GC_* is the work function difference, ∅*_F_* is the Fermi potential and *Q_ox_* is positive charge density at the gate oxide/Si substrate interface.

*Q_B_* is the depletion region charge developed in the substrate.

**Figure 3:**
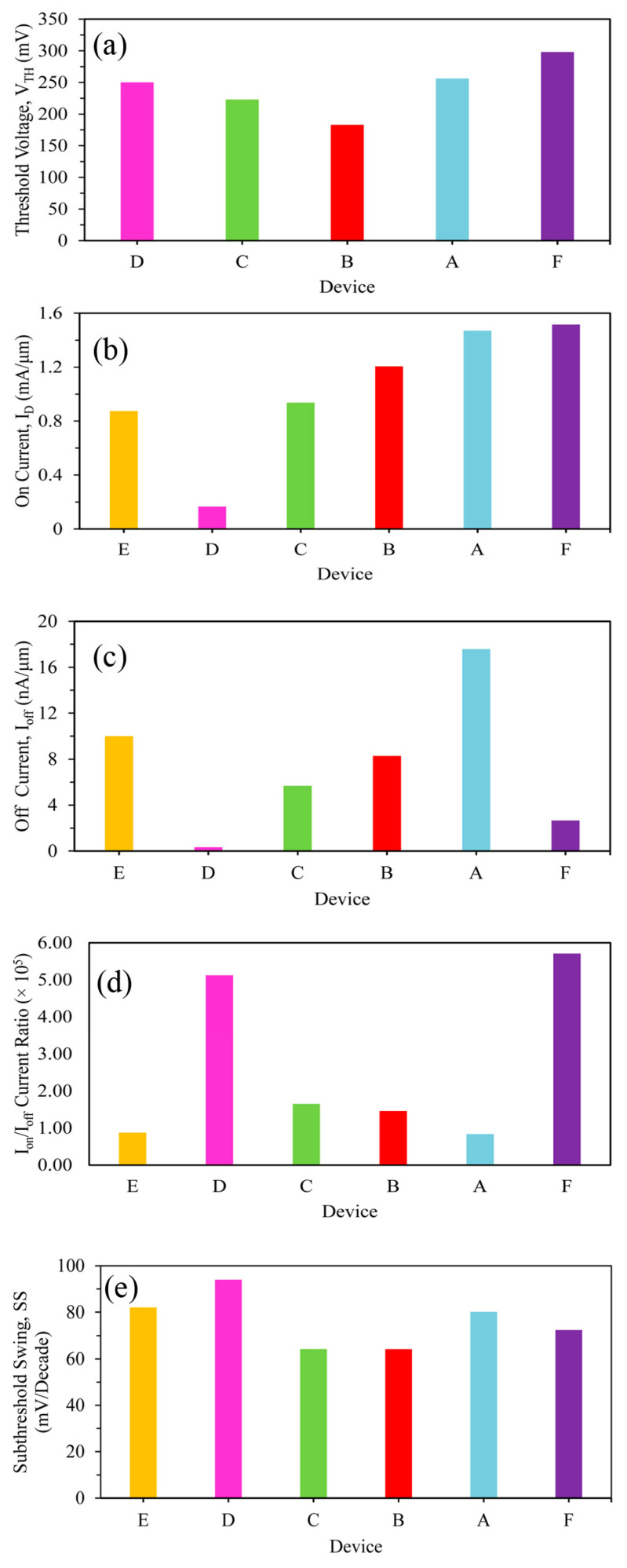
(**a**) Comparison of V_TH_ of devices A, B, C, D and F. (**b**) Comparison of I_on_ and (**c**) I_off_ current of devices A–F. (**d**) I_on_/I_off_ ratio of devices A–F. (**e**) Comparison of subthreshold swing of devices A, B, C, D, E and F.

The threshold voltage and on currents of devices A to F are compared in Figure 3a,b, which clearly show that the 10 nm HOI high-k gate stacked GAAFET has a superior on current of 1.51 mA/μm in comparison with the 22 nm HOI GAAFET with an on current of 1.21 mA/μm, and 0.166 mA/μm for the 10 nm SOI TG FinFET. The on current of device F is far ahead of the requirements of the on currents of IRDS 2022 specifications at 0.874 mA/μm owing to the application of the tri-layered strained channel delivering a ballistic transport phenomenon with minimum scattering effect, which drastically increases the drain current of the device.

The n-channel FET is considered to be in the off state if the gate to source voltage $V_{GS}$ is less than the $V_{TH}$. The leakage current is named subthreshold current, which flows under the condition $V_{GS}$ < $V_{TH}$. A lower subthreshold current subscribes to diminished off current I_off_.

For better device performance, the static power should be minimized, and the value of I_off_ must be a low value.

The expression for the subthreshold current [28] is

(4) $$I_{d(subthreshold)}=\frac{qD_{n}Wx_{c}n_{0}}{L_{g}}\cdot e^{\frac{{q\emptyset }_{r}}{KT}}\cdot e^{\frac{q({A.V}_{GS}+{B.V}_{DS})}{KT}}$$

The leakage currents of the different devices are depicted in Figure 3c. The leakage current of device F is 2.66 nA/μm, which is well below 10 nA/μm, the requirements of IRDS 2022. Similarly, the leakage currents of devices A–C are determined to be 17.59, 8.27 and 5.68 nA/μm, respectively, and, therefore, are discarded. Since device D is a TG FinFET employing only a silicon channel, the leakage current is determined to be considerably smaller. But, since the on current of device D is also small, it becomes obvious that the leakage current is also diminished in device D.

The I_on_/I_off_ current ratios of all the devices are specified in Figure 3d, which are a measure of the switching speeds of the devices. The I_on_/I_off_ current ratio of devices F employing a gate stack arrangement is the highest at 5 × 10^5^, while it is only 0.84 × 10^5^ for the same 10 nm channel length GAAFET device A but employing 1 nm of SiO_2_. The I_on_/I_off_ current ratios of devices B, C and D are determined to be 1.46 × 10^5^, 1.65 × 10^5^ and 5.12 × 10^5^, while it is 0.87 × 10^5^ for the IRDS 2022 specifications.

Thus, device F, with a higher on-current and I_on_/I_off_ current ratio along with acceptable leakage currents, clearly outperforms all the other devices.

The leakage current is given in the below equation:

(5) IleaknA=100WFinLCh10VTHSS

where $W_{Fin}$ is the width and *L_Ch_* is the channel length, $V_{TH}$ being the $V_{TH}$ and SS represents the subthreshold swing.

The developed 10 nm strained silicon GAAFET device F has a subthreshold swing (SS) of 72.42 mV/decade, which is much less compared with 80.15 mV/decade of device A. The subthreshold swings of the 22 nm strained silicon GAAFET device B, 22 nm silicon GAAFET device C, 10 nm TG FinFET device D and IRDS specifications device E are 64 mV/decade, 73 mV/decade, 94 mV/decade and 82 mV/decade, respectively. A comparison graph is shown in Figure 3e. The subthreshold swing (SS) value of device F is considerably lower than the 10 nm SOI FinFET as well as the IRDS 2022 requirements. Similarly, the DIBL of device F is 56.24 mV/V, which is lower than that of device A and B at 62.42 and 60.55 mV/V while being on par with the DIBL of device C at 54.37 mV/V. Therefore, considering all the parameters, the performance of the 10 nm channel length GAAFET with stacked high k and tri-layered strained silicon channel, termed device F, is superior.

Characteristic analysis of all the devices is performed, compared with each other, and a detailed analysis of V_TH_, I_on_/I_off_ current t ratio and subthreshold swing is shown in Table 3. It is observed from Table 3 that the I_on_ current of the 10 nm channel length HOI GAAFET using gate stack combination of SiO_2_ and HfO_2_ (device F) shows ~8-times increase in comparison with the 10 nm SOI TG FinFET (device D). Device F is also observed to provide ~25% increase in I_on_ current when compared to the 22 nm channel length HOI GAAFET (device B). Similarly, the I_on_/I_off_ current ratio of device F shows an enrichment of ~2.9- and ~5.8-times in comparison to that of the 22 nm channel length HOI GAAFET (device B) and 10 nm channel length GAAFET using 1 nm of SiO_2_ (device A), respectively. The I_on_/I_off_ current ratio of device F is observed to be similar to that of the 10 nm SOI TG FinFET employing high-k dielectrics as the gate oxide. The comparison of the subthreshold swing yields a smaller value for device F in comparison to devices A and D, concluding that device F has superior performance among all the existing devices and, hence, may be considered as the device for the future.

**Figure 4:**
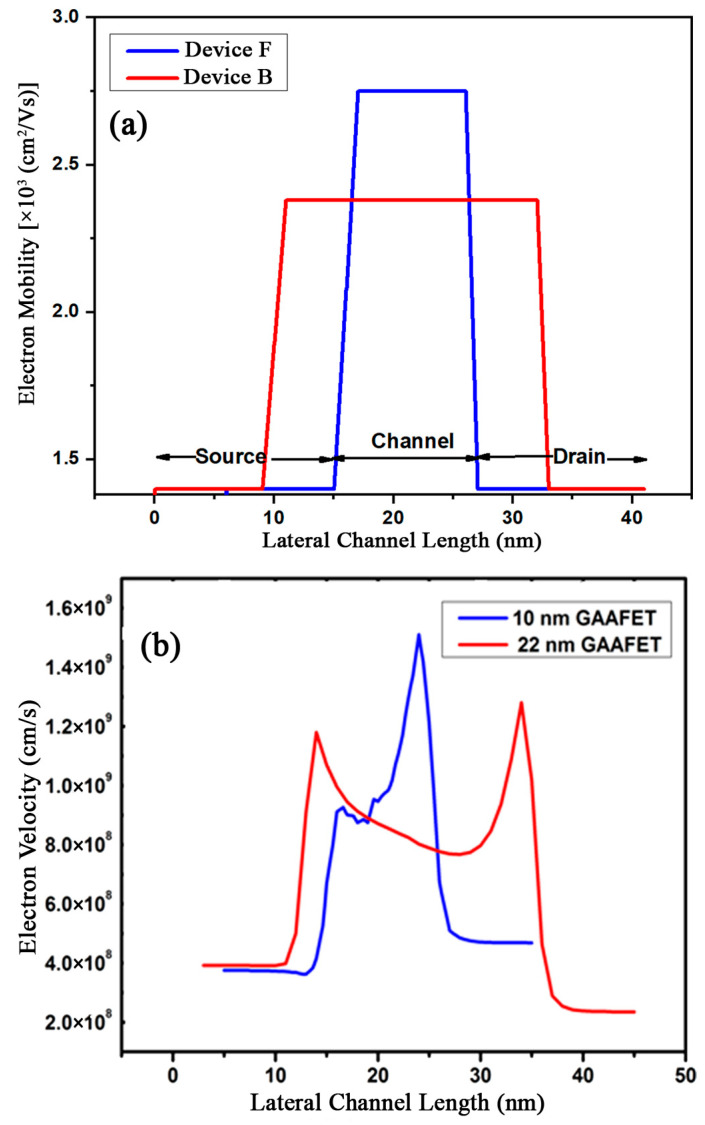
(**a**) Comparison of electron mobility for the advanced GAAFET with high-k (device F) and the existing GAA FET (device B). (**b**) Electron velocity comparison of devices F and B.

When a strained silicon channel is induced in a semiconductor device with a very short channel length, the energy gap becomes very thin, and the atoms are tightly packed in all three directions. The difference in the lower sub-band and upper sub-band reduces the transverse effective mass. The e^−^ mobility comparison is shown in Figure 4a.

When the gate biasing is provided to the four gates, the drift and electron velocity along the channel length cause high inversion by the coupling of four gates at the middle of the three-layered channels, which is the strained SiGe layer. This layer is formed inside the two strained silicon layers, which have more majority charge carriers in the strained SiGe layer. With an increased electron velocity, more and more charge carriers will move from source to drain, which results in ballistic transport in the device with less intervalley scattering and a large amount of drift velocity, as shown in Figure 4b.

**Figure 5:**
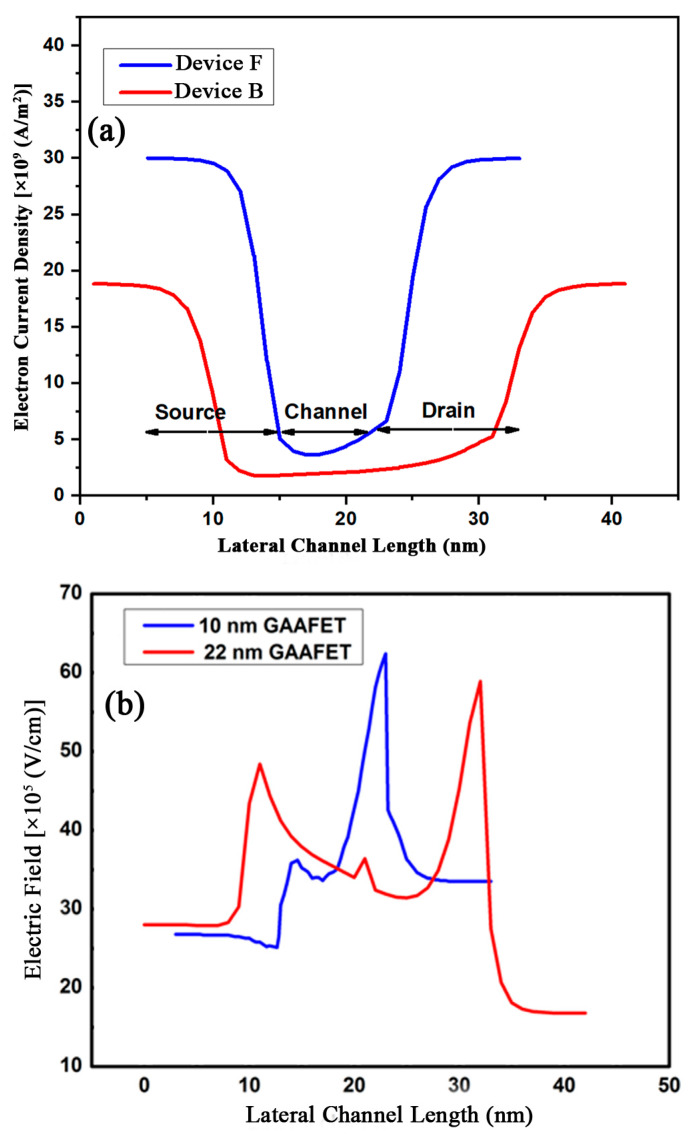
(**a**) Study of electron current density of devices F and B. (**b**) Electric field comparison of 10 nm and 22 nm HOI GAAFETs.

**Figure 6:**
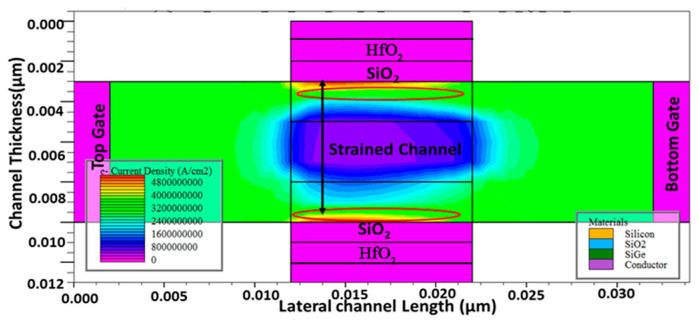
Contour diagram of electron current density across the cross-section of the 10 nm HOI GAAFET.

The lateral electric field and the electric potential have an influence on the SCEs in the devices. When the strained silicon channel is introduced in these short channel devices, the drain to gate tunneling current is avoided. This results in more charge carriers moving in shorter channels ballistically with minimal scattering. This leads to high electron current density, as observed in Figure 5a and Figure 6. The contours of the electron density in Figure 6 clearly show the electron transport though the s-Si layers, while it is negligible in the middle s-SiGe layer.

The electric field from Figure 5b shows the variations in the device along the lateral direction. This happens when 1 V of the supply voltage is biased with the gate and drain terminal. The electric field at the drain and source is different in both regions, which is due to the V_DS_. As all three layers of the channel have the same doping concentration, the electron velocity is varied at the drain to channel interface and source to channel interface because the doping concentration is higher at the source/channel interface.

**Figure 7:**
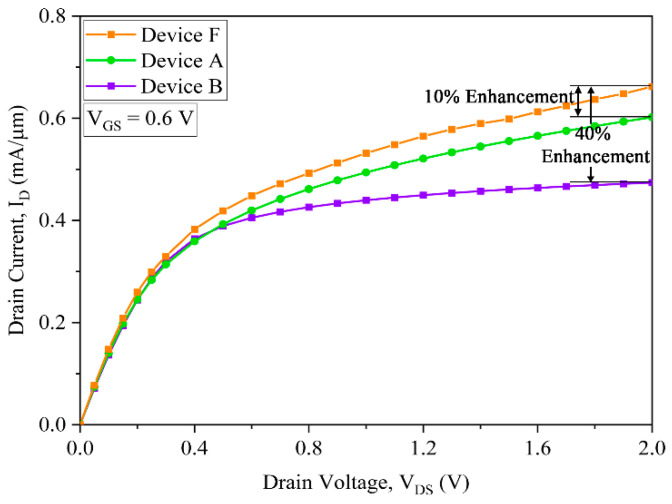
I_D_–V_DS_ characteristic comparison of devices A, B and F.

The I_D_–V_DS_ plot is shown in Figure 7, which shows the 22 nm strained silicon (device B) and 10 nm strained silicon (devices F and A) characteristics. It is clearly observed that the 10 nm strained silicon device F shows better performance than device A as well as the 22 nm strained silicon GAAFET device B.

It is also inherent from Figure 7 that the enhancement in the drain current of device F is ~80% and ~80% in comparison with that of devices A and B. The increase in the current due to the biaxial strain is instigated in the channel, and, hence, the mobility is enhanced in the GAAFET.

## 4. Conclusions

The 10 nm channel length strained silicon GAAFET is implemented with the three layers of strained silicon in the channel, which induces the ballistic transport effect and optimizes the device for enhanced performance. The gate oxide is initially considered as 1 nm of SiO_2_ (device A), which is then enhanced by replacing the SiO_2_ layer with a gate stack of SiO_2_ and HfO_2_ (device F). The various electrical properties, such as SS, V_TH_, and I_on_/I_off_ ratios, of device F are characterized and compared with device A, as well as 22 nm HOI GAAFET (device B), 22 nm SOI GAAFET (device C), 10 nm SOI FinFET (device D) and IRDS 2022 specifications (device E). The on current of device F is 1.51 mA/µm, while the I_off_ current is 2.66 nA/µm, leading to a higher I_on_/I_off_ current ratio of 5.71 × 10^5^, which means this device has superior performance. The SS is determined to be 72.42 mV/decade, and the DIBL is 56.24 mV/V, which characterizes the improved short channel effects. The study of quantum effects via the electron velocity and electron mobility graphs and contours shows the electron confinement in the strained silicon layer, which results in increased drain current. This is attributed to the lower amount of phonon scattering, leading to ballistic transport in the channel region.

## Figures and Tables

**Table 1:** Device dimension specifications.

Variable	Dimensions
Source/Drain/Channel Length	10 nm
Channel Thickness (Upper s-Si)	2 nm
Channel Thickness (Middle s-SiGe)	3 nm
Channel Thickness (Lower s-Si)	2 nm
Drain/Source Conc.	5 × 10^19^ cm^−3^
EOT	0.7 nm
Gate Work Function	4.64 eV

**Table 2:** Device design specifications.

Device	Characteristics
A	10 nm Heterostructure on Insulator(HOI) Gate-All-Around FET (1 nm SiO_2_)
B	22 nm Heterostructure on Insulator (HOI) Gate-All-Around FET [24]
C	22 nm Silicon on insulator (SOI) Gate-All-Around FET [24]
D	10 nm Silicon on insulator (SOI) Trigate FinFET [25]
E	IRDS 2022 specifications [12]
F	10 nm Heterostructure on Insulator (HOI) Gate-All-Around FET (Gate Stack, 0.7 nm EOT)

**Table 3:** Device characteristics.

Device	V_TH_ (mV)	I_on_ (mA/μm)	I_off_ (nA/μm)	I_on_/I_off_ Ratio (×10^5^)	SS (mV/dec)	DIBL (mV/V)
A	256	1.47	17.59	0.84	80.15	62.42
B	183	1.206	8.27	1.46	64.19	60.55
C	223	0.937	5.68	1.65	64.24	54.37
D	250	0.166	0.32	5.12	94	Not Available
E	Not Available	0.874	10.00	0.87	82	Not Available
F	298	1.516	2.66	5.71	72.42	56.24

## Data Availability

All data included in article.
